# Novel Pharmacological Treatment Options of Steroid-Refractory Graft-versus-Host Disease

**DOI:** 10.1155/2023/9949961

**Published:** 2023-12-06

**Authors:** Iuliia Kovalenko, Tabinda Saleem, Mitali Shah, Sara Seyedroudbari, Konstantin Golubykh, Rimsha Ali, Taaha Mirza, Babray Laek, Ahsan Wahab, Asmi Chattaraj, Ekaterina Proskuriakova, Chandi Garg, Rafiullah Khan

**Affiliations:** ^1^UPMC Harrisburg, Harrisburg, PA, USA; ^2^UPMC Presbyterian/Montefiore Hospitals, Pittsburg, PA, USA; ^3^Drexel University College of Medicine, Philadelphia, PA, USA; ^4^UPMC McKeesport, Pittsburg, PA, USA; ^5^Levine Cancer Institute, Charlotte, NC, USA; ^6^Mount Sinai Hospital, Chicago, IL, USA; ^7^The Christ Hospital Network, Cincinnati, Ohio, USA

## Abstract

**Background:**

Graft-versus-host disease (GVHD) is a potentially fatal complication of allogeneic hematopoietic stem cell transplant. The mainstay of treatment is corticosteroids, which are ineffective in 30–50% of cases. Steroid-refractory GVHD (SR-GVHD) confers a poor prognosis, with high mortality rates despite appropriate therapy. While there is no reliable treatment for SR-GVHD, a variety of novel therapeutic options are slowly emerging and have yet to be examined simultaneously.

**Objectives:**

This review evaluates the potential of novel therapeutic options, as well as their efficacy and safety, for the treatment of SR-GVHD. *Study Design*. The literature search was conducted in PubMed, Cochrane, and Embase, employing MeSH terms and keywords. The studies had to be prospective phases 1, 2, or 3. We excluded retrospective and nonoriginal studies.

**Results:**

While the only approved drug for acute GVHD is ruxolitinib with an impressive overall response rate of 73.2% and a complete response of 56.3%, several monoclonal antibodies and other agents are currently under investigation, offering promising results. These include anti-CD2, anti-CD147, IL-2 antagonist, a mixture of anti-CD3 and anti-CD7 antibodies, anti-CD25, monoclonal antibody to a4b7 on T-cells, anti-CD26, pentostatin, sirolimus, denileukin diftitox, infliximab, itacitinib, and alpha-1 antitripsin. However, the toxicities associated with these novel drugs need further investigation. For chronic GVHD, approved options include ruxolitinib with an ORR of up to 62%, ibrutinib with an ORR of up to 77%, and belumosudil with an ORR of up to 77%. Meanwhile, emerging treatments include tyrosine kinase inhibitors such as nilotinib, rituximab, and low-dose IL-2, as well as axatilimab and pomalidomide.

**Conclusion:**

While their efficacy needs to be better evaluated through large-scale, multicenter, randomized clinical trials, these novel agents show potential and could provide a better alternative for SR-GVHD treatment in the future.

## 1. Introduction

**Figure 1:**
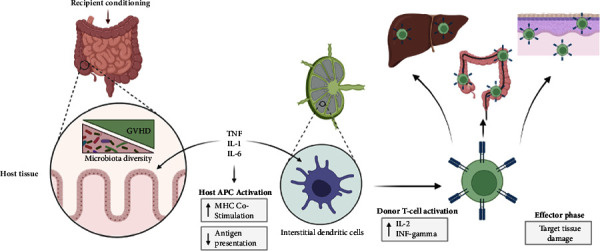
Pathophysiology of graft-versus-host disease. TNF = tumor necrosis factor alpha; IL = interleukin; INF = interferon.

Graft-versus-host disease (GVHD), a reaction of donor immune cells against host tissue, is a potentially fatal complication of allogeneic hematopoietic stem cell transplant [1]. The mainstay of treatment is corticosteroids, which are ineffective in 30–50% of cases. Steroid refractory GVHD (SR-GVHD) confers poor prognosis, and treatment beyond conventional therapy remains a largely untapped topic, with a variety of novel therapeutic options slowly emerging as promising answers. In the context of SR-GVHD, understanding its underlying mechanisms is crucial for developing new treatments. SR-GVHD emerges when T lymphocytes from the donor perceive the recipient's cell antigens as alien, leading to a cascade of cell activation and cytokine secretion (Figure 1). This process inflicts damage on the recipient's tissue cells, exacerbating the harm already caused by the primary illness and immunosuppressive treatments. Central to SR-GVHD's pathogenesis are histocompatibility disparities and the failure of current immunosuppression strategies. Notably, steroids trigger a response in toll-like receptor 4-activated monocytes, fostering the growth of proinflammatory T-17 helper and cytotoxic T-17 cells. These cells eventually become resistant to the apoptotic and cytokine suppression effects of steroids [2]. Consequently, there is an acute need to explore new pharmacological interventions that either effectively suppress the host's immune response or target the action of these T-17 cells. Such advancements are vital to address the complexities and challenges of treating SR-GVHD. We examine recent studies that assess the role of new agents in the treatment of SR-GVHD.

## 2. Methods

A comprehensive reference search was done across the following databases: PubMed, Cochrane, and Embase. MeSH terms and keywords were employed to capture a variety of drugs that have been studied for SR-GVHD. The following concepts were searched using subject headings and keywords as needed: “steroid refractory graft-versus-host disease,” “acute steroid refractory graft-versus-host disease,” “chronic steroid refractory graft-versus-host disease,” “treatment of steroid refractory graft-versus-host disease,” “treatment of chronic steroid refractory graft-versus-host disease,” “treatment of acute steroid refractory graft-versus-host disease,” “ruxolitinib in steroid refractory graft-versus-host disease.” Our study did not involve animal or human studies and was exempted from Institutional Review Board approval. In determining eligibility for our review, we established several inclusion criteria. The studies had to be prospective phase 1, 2, or 3 and studies had to be reported in English. We excluded retrospective and nonoriginal studies.

### 2.1. Novel Drugs in the Treatment of Acute SR-GVHD

#### 2.1.1. Ruxolitinib

Ruxolitinib, a JAK1 and JAK2 kinase inhibitor, is used for the treatment of acute SR-GVHD (SR-aGVHD). It was approved by the FDA based on the results of the REACH-1 trial, a prospective, multicenter, open-label, single-cohort, phase II trial including 71 patients, 12 years of age and older, with SR-aGVHD [3]. Patients received a starting oral dose of ruxolitinib of 5 mg twice daily (BID), with an increase to 10 mg BID after 3 days in the absence of cytopenia. The median follow-up was 156 days. The overall response rate (ORR) was 73.2%, with a complete response (CR) in 56.3%. The median survival was 7.6 months.

#### 2.1.2. Monoclonal Antibodies in the Treatment of Acute SR-GVHD

Although IL-2 receptor *α* (IL-2R*α*) antagonists have not shown promising long-term results in SR-aGVHD treatment, Bordigoni et al. explored daclizumab as a second-line agent in 62 patients with SR-aGVHD [4]. In a prospective, single-center, phase II study, patients with SR-aGVHD received daclizumab. A total of 68.8% of patients achieved complete response, while 21.3% achieved partial response. Patients with low-grade SR-aGVHD, especially those limited to the skin or GI tract, had the best overall response rate. Median follow-up was 44 months, and the reported four-year event-free survival was 54.6%.

A mixture of anti-CD3 and anti-CD7 antibodies separately conjugated to recombinant ricin A, termed anti-CD3/CD7 immunotoxin (CD3/CD7- IT), showed promising results as a third-line therapy for SR-aGVHD in a prospective, single-arm, phase I/II dose-escalating study [5]. A total of 20 patients with SR-aGVHD between ages 18 and 74 received immunotoxin. On day 28, the overall response rate was 60%, and the rate of complete response was 50%. Reported 6-month overall survival was 60%, including 64% of patients classified as “high-risk.” Two-year overall survival was 35%, improved from historical controls of 16.7%.

Inolimomab, an anti-CD25 mAb, has shown encouraging results for short-term survival rates in phase II trials for SR-aGVHD. Socié et al. conducted a randomized, multicenter, open-label, phase III trial comparing inolimomab to standard care in 100 adults with SR-aGVHD [6]. Reported one-year overall survival was 28.5% in the treatment arm compared to 21.5% in the control arm.

Vedolizumab, a humanized monoclonal antibody approved by the FDA for the treatment of ulcerative colitis and Crohn's disease, has been evaluated in gastrointestinal SR-aGVHD. The drug inhibits the interaction of a4b7 on T-cells with their ligand MAdCAM-1 on the endothelium of venules, thereby suppressing the local immune system in the gastrointestinal tract. In a prospective study by Mehta et al. 20 patients with gastrointestinal SR-aGVHD were treated with vedolizumab. Reported ORR and CR at 56 days were 25% and 20%, respectively, with a 6-month OS of 35%. The most commonly reported AEs included infections and liver enzymes elevation [7].

Monoclonal antibody against CD26, an antigen that is expressed by activated T-cells and has costimulatory functions playing a role in the development of the GVHD, has been also considered a potential treatment option. Bacigalupo et al. have analyzed the efficacy of the begelomab, a monoclonal antibody against CD26, based on two prospective studies (group I) and one compassionate use study (group II). They have shown that the ORR was 75% and 61% while the CR was 11% and 12% in groups I and II, respectively. Reported one-year OS was 50% and 30% in groups I and II, respectively. Although all patients developed AEs, severe toxicities were observed from one to three patients and included bronchopneumonia (25%), dyspnea (16.6%), bacterial sepsis, convulsions, acute respiratory failure, multiorgan failure, and *E. coli* infection with renal failure (0.06%, 1 patient) [8].

Visilizumab is a humanized non-FcR-binding anti-CD3 mAb that induces apoptosis selectively in activated T-cells. Carpenter et al. present the results of a multicenter, single-arm, phase II trial with 44 participants in which 86% had overall grade III or IV aGVHD at study entry [9]. At day 42, the overall response rate was 32% and the complete response rate was 14%. Reported overall survival at 180 days was 32%, while the median survival was 539 days.

#### 2.1.3. Novel Agents in the Treatment of Acute SR-GVHD

Pentostatin, an adenosine deaminase inhibitor, is known to be beneficial in conditioning regimens to prevent GVHD. A phase I dose-escalation study assessed its effects on SR-aGVHD in 23 patients between 6 months and 63 years old. A total of 63% of patients achieved a complete response, while 14% achieved a partial response [10]. Median survival after therapy initiation was 85 days.

Sirolimus, an mTOR inhibitor, has shown efficacy in preventing renal transplant rejection and rejection refractory to other therapy. In a pilot study, Benito et al. studied sirolimus in 21 patients with SR-aGVHD [11]. The reported overall response rate was 57%, with a complete response in a 24%. An unfavorable toxicity profile suggests further dose optimization studies are warranted. The true therapeutic potential of sirolimus may be better determined if it is used earlier in the disease course.

Denileukin diftitox, a protein composed of IL-2 fused to diphtheria toxin, possesses cytotoxic activity against activated lymphocytes expressing high-affinity IL-2 receptors. Ho et al. explored the effects of denileukin diftitox in 30 patients with SR-aGVHD in a single-center, multicohort, prospective, and phase I study [12]. The reported overall response rate was 71%, with complete response in 50% and a partial response in 21%. Patients achieved an overall survival rate of 33% during the observation period from 6.3 to 24.6 months. Median survival was 7.2 months.

Infliximab, a TNF-alpha inhibitor, has shown efficacy in the treatment of SR-aGVHD, with response rates ranging from 59% to 67% [13]. Couriel et al. conducted a prospective, single-center, open-label, randomized, phase III study that evaluated infliximab earlier in the treatment course of SR-aGVHD. A total of 63 participants were randomized to either 2 mg/kg/day methylprednisolone or infliximab plus methylprednisolone. On day 28, the reported overall response rate was 62% and 58% in the treatment and control groups, respectively (*p*=0.03), with equal complete response between the two groups. Patients achieved similar overall survival rates of 17% and 28% in the treatment and control groups, respectively (*P*=0.4).

Itacitinib is a JAK1-selective inhibitor that has demonstrated efficacy against aGVHD in preclinical models. In an open-label, parallel-cohort, multicenter, and phase I trial by Schroeder et al., 29 participants with SR-aGVHD received itacitinib [14]. The reported overall response rate on day 28 was 75% and 66.7% in patients who received 200 mg daily and 300 mg daily, respectively. Achieved overall survival estimates at 6 and 12 months were 58.6% and 48.3% for all participants.

Alpha-1 antitrypsin (AAT), a serine protease inhibitor with anti-inflammatory properties, has been shown to decrease the expression of proinflammatory cytokines which play a role in GVHD such as IL-1, tumor necrosis factor, and IL-32, in preclinical models [15]. Marcondes et al. have evaluated the efficacy of AAT in a phase I/II open-label single-center study where they administered AAT to 12 patients with SR-aGVHD. Reported ORR was 66.5% with CR of 33.3%, while OS was 50% at >104 to >820 days. Authors did not observe any clinically relevant toxicities [16]. All the data on the novel drugs in the treatment of acute SR-GVHD is summarized in Table 1.

### 2.2. Novel Drugs in the Treatment of Chronic SR-GVHD

#### 2.2.1. FDA-Approved Treatments

Zeiser et al. conducted a multicenter, randomized, open-label phase 3 trial of oral ruxolitinib in 309 patients ages 12 and older with chronic SR-GVHD (SR-cGVHD) [17]. A total of 154 patients were assigned to the ruxolitinib group and 155 to the control group. Reported overall response rates at 28 days were 62% and 39% in the ruxolitinib and control groups, respectively (*p* < 0.001). Overall response rates at 56 days were 40% and 22% in the ruxolitinib and control groups, respectively (*p* < 0.001). Patients were not stratified based on the organ involvement but improvement of the symptoms was observed in all patients based on the odds ratio. The median failure-free survival and median overall survival were significantly higher in the ruxolitinib group compared to control group (5 months versus 1 month and 11.1 months versus 6.5 months, respectively). As a result of this trial, ruxolitinib was approved by FDA to treat patients 12 years of age and older with cGVHD who failed one or two lines of systemic therapy.

Ibrutinib, an oral Bruton tyrosine kinase inhibitor, was approved for the treatment of SR-cGVHD in pediatric patients 1 year of age or older after failure of 1 or more lines of therapy, as a result of the iMAGINE trial [18]. This phase 1/2 multicenter, international study included 59 patients, ages 1 to 22 years, with moderate to severe cGVHD, among whom 47 patients had relapsed or refractory disease. Median duration of follow-up for the relapsed/refractory group was 20.6 months. The reported overall response rate was 77% in the relapsed/refractory group, with partial response in 72% and complete response in 4%. Responses were observed in patients with various organs involvement regardless of the baseline status with no subgroup analysis performed. Estimated duration of response and event-free survival at 18 months in relapsed/refractory group were 58% and 49%, respectively. Reported overall survival at 18 months was 91% in the relapsed/refractory group. Overall, ibrutinib showed substantial efficacy and a tolerable safety profile, prompting its approval for treatment of relapsed/refractory cGVHD in children.

Studies have shown the clinical benefit of ibrutinib for SR-GVHD in adults as well. Noriko et al. conducted an open-label, single-arm multicenter study of ibrutinib in Japanese patients greater than 12 years old with SR-cGVHD [19]. The median duration of treatment was 9.63 months. Ibrutinib was rapidly absorbed, with a median time to reach maximum plasma concentration of 4 hours. The overall response rate was 73.7%, with complete response in 10.5%. The median daily corticosteroid dose requirement decreased 0.06 mg/kg/d from baseline. Although the median duration of response was not reached during the observation period, estimated duration of response was 84.6% and 74% at 6 and 9 months, respectively. The best responses were observed in liver (100%), lower and upper gastrointestinal tracts (66.7% and 60%, respectively), followed by the musculoskeletal system (40%), and skin (35.7%). As a result of this trial, ibrutinib was approved by FDA to treat patients 12 years, and older with cGVHD who failed one or more lines of systemic therapy.

Belumosudil, an oral selective Rho-associated coiled-coil–containing protein kinase 2 (ROCK2) inhibitor, acts through downregulation of STAT3 phosphorylation leading to inhibition of T-helper 17 expression affecting fibrotic pathways. This drug has attracted researchers' attention due to its unique pathophysiological properties. In a phase 2 randomized multicenter registration trial called the Rockstar trial by Cutler et al., a total of 132 patients with previously treated cGVHD received belumosudil 200 mg daily or twice daily [20]. The reported overall response rate was 74% and 77% in daily and BID-dosed groups, respectively. At 12 months, in the 200 mg daily group, complete and partial response rates were 6% and 68%, respectively, while in the 200 mg BID group complete and partial response reached 3% and 73%, respectively. Overall, the duration of response was 54 weeks, while failure free survival at 12 months was 56%. Reported 2-year overall survival rate was 89%. Organ-specific subgroup analysis showed the best ORR in the joints/fascia (71%), upper and lower gastrointestinal tract (69% and 52%, respectively), as well as mouth (55%), followed by eyes (42%), skin (36%), and lungs (26%). As a result of the trial, Belumosudil was approved by the FDA for adult and pediatric patients 12 years of age and older with cGVHD after failure of at least two prior lines of systemic therapy.

#### 2.2.2. Tyrosine Kinase Inhibitors in the Treatment of SR c-GVHD

Nilotinib is a tyrosine kinase inhibitor that targets the same receptors as imatinib (a known agent with clinical activity in cGVHD) but with different affinities. A proposed mechanism of action of imatinib is the inhibition of autoantibody-mediated platelet-derived growth factor receptor (PDGFR) activation. Chen et al. conducted a phase I/II trial with 33 participants to assess the safety, clinical response, and pretreatment anti-PDGFR alpha chain (anti-PDGFRA) in patients with cGVHD [21]. Reported failure-free survival was 50% at 6 months and 23% at 1 year. The median change in prednisone equivalent dose from baseline to 3, 6, and 12 months was −0.01 mg/kg/day (not significant), −0.06 mg/kg/day (*P* < 0.05), and −0.08 mg/kg/day (not significant), respectively. Of the 26 patients at 3 months, 12 (46%) were scored as partial responders and 18 (54%) as nonresponders with either no change (*n* = 2) or progression (*n* = 16) of the disease. Responses were most frequently seen in mouth, skin, and joints with symptomatic improvement also observed in the eye and upper and lower gastrointestinal tracts.

#### 2.2.3. Novel Treatments for SR c-GVHD

Macrophages that depend on the colony-stimulating factor 1 receptor (CSF-1R) play a significant role in promoting cGVHD fibrosis. Recent preclinical studies have shown that targeting these macrophages can effectively reduce cGVHD. In this context, axatilimab, a humanized monoclonal antibody, demonstrates promise by inhibiting CSF-1R signaling and controlling macrophage proliferation. Conducted by Kitko and colleagues, this medication underwent a phase I/II open-label study [22]. The research involved 40 participants aged six years and above, all of whom had active cGVHD and had undergone at least two previous systemic therapies. The treatment involved administering axatilimab at a dose of 3 mg/kg every four weeks. During the study, two cases of dose-limiting toxicities were noted with a biweekly dosage of 3 mg/kg. By the first day of the seventh cycle, the overall response rate (ORR) reached 82%, and the highest ORR observed at any point was 69%. The highest response rate was observed in joints/fascia (61%), lung (31%), and skin (14%).

Hedgehog signaling pathways contribute to the process of tissue fibrosis, one of the key pathophysiological mechanisms of cGVHD. DeFilipp et al. conducted a phase 1 clinical trial of sonidegib, a selective antagonist of the hedgehog coreceptor smoothened, for the treatment of SR-GVHD [23]. Sonidegib was administered to 17 patients for up to 12 cycles of 28 days each, using 3 doses: 200 mg/day (level 1), 400 mg/day (level 2), or 600 mg/day (level 3). The median number of cycles completed was 6, ranging from 0 to 12, with one dose-limiting toxicity observed (grade 3 creatinine phosphokinase elevation). Immunohistochemical evaluation of skin biopsies showed decreased protein expression of hedgehog signaling pathway molecules after therapy. A total of 8 patients (47%) achieved partial response in skin or sclerodermatous disease. Six patients (36%) had no response. 3 (17%) were not able to be evaluated. Subgroup analysis for various organs has not been performed. The maximum tolerated dose was not reached.

Cutler et al. conducted a phase 1/2 study of anti-B-cell therapy with rituximab, as B-cells have been implicated in the pathophysiology of cGVHD [24]. A total of 21 patients were treated with at least one cycle of rituximab 375 mg/m^2^/week for 4 consecutive weeks. The authors report that 68% of patients had a corticosteroid dose reduction of at least 50%. Reported complete and partial response rates were 10% and 60%, respectively. The subgroup analysis of the efficacy has not been performed but according to symptoms scale 60% of patients reported cutaneous symptoms improvement while 64% of the patients reported musculoskeletal symptoms improvement.

Low-dose interleukin-2 (IL-2) therapy generates a rapid rise in plasma levels of IL-2 and the proliferation of various regulatory T-cells. The levels, however, decrease over time despite continuing therapy. Whangbo et al. tested whether IL-2 dose escalation at the time of anticipated drops in plasma levels could enhance regulatory T-cell expansion [25]. They conducted a phase 1 clinical trial with 10 adults and 11 children with SR-cGVHD. In the pediatric patients, 82% had a partial response and 18% had stable disease/mixed response. Of the 7 evaluable adult patients, 28.5% had a partial response, 28.5% had stable disease/mixed response, and 43% had progressive disease. These findings are supported by the study performed by Kim et al., who combined 5 phase 1 and 2 clinical trials to analyze organ-specific response of SR-cGVHD to IL-2. Among 105 evaluated patients ORR was 48.6% and 53.3% at 8 and 12 weeks, respectively. Reported organ-specific response was highest in liver (66.7%), gastrointestinal tract (62.5%), skin (36.4%), joint/muscle/fascia (34.2%), and lung (19.2%) [26].

Pomalidomide, an immune-modulating drug structurally related to thalidomide (an effective therapy for severe cGVHD in certain cases), is currently approved for the treatment of multiple myeloma. It increases CD4+ T-cells, suppresses T-helper type 2 cells, acts directly on B-cell proliferation, and stimulates IL-2 and soluble IL-2 receptor production. Curtis et al. conducted a randomized phase 2 clinical trial of pomalidomide in 34 patients with moderate-to-severe SR-cGVHD [27]. In the 24 evaluable patients, the overall response rate was 67% at 6 months, with no complete responses and no differences between the dosing groups. The highest response rates were observed in the joint/fascia, gastrointestinal tract, mouth, and skin.

**Figure 2:**
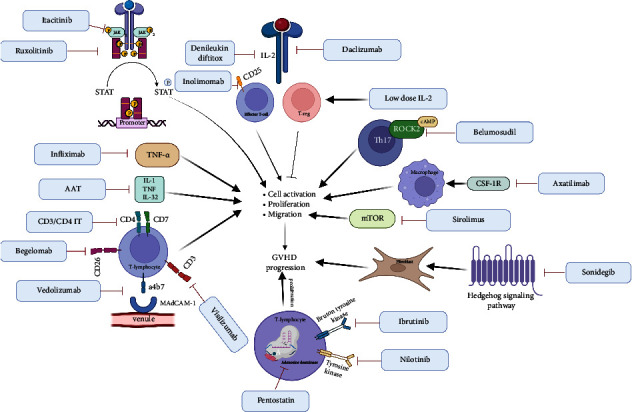
Mechanism of action of various novel therapies in GVHD. TNF = tumor necrosis factor; AAT = alpha antitrypsin; IL = interleukin; TNF = tumor necrosis factor; T-reg = T-regulatory cell; CSF-1R = colony-stimulating factor 1 receptor.

Figure 2 summarizes all novel pharmacological therapies for acute and chronic SR-GVHD.

## 3. Discussion

Between 30% and 70% of patients who receive allogeneic hematopoietic stem cell transplants develop some degree of GVHD, with steroids typically being the first line of treatment. However, 35% to 50% of GVHD cases do not respond to steroid treatment. There is currently no consensus on the optimal treatment regimen for SR-GVHD.

GVHD occurs when donor-derived T lymphocytes recognize recipient cell antigens as foreign, triggering cell activation and cytokine release that destroy host tissue cells. This insult can further exacerbate the already compromised host tissue caused by the underlying disease and immunosuppressive therapy. The theory behind the pathogenesis of SR-GVHD involves histocompatibility differences and inadequate immunosuppression. Steroids induce a toll-like receptor 4-activated monocyte response that promotes the development of proinflammatory T-17 helper and cytotoxic T-17 cells, which later become resistant to steroid-induced apoptosis and suppression of the cytokine response. Therefore, the current focus of drugs for SR-GVHD treatment is either effective host immunosuppression or activity against these T-17 helper and cytotoxic T-cells [2].

Ruxolitinib is currently the only FDA-approved drug for the treatment of aGVHD, but recent studies have shown promising results for other drugs such as monoclonal antibodies. Daclizumab, an anti-CD25 mAb, has shown promising results, but its adverse events profile has not been fully reported. Anti-CD3/CD7-IT has also shown promising results, with cytopenia being a major concern.

Several drugs have shown promising results for the treatment of aGVHD. Monoclonal antibodies such as daclizumab and visilizumab, which target CD25 and CD3, have shown modest activity against aGVHD. Pentostatin, a nucleoside-inhibitor, had an overall response rate of 76% and complete response in 63%, with a favorable adverse events profile except for two patients who developed grade 5 infections. Denileukin diftitox had an overall response rate of 71%, and complete response in 50%, with longer median survival. Sirolimus, an mTOR inhibitor, had an overall response rate of 57%, with complete response in 24%, with rare severe adverse events. Combination treatments such as infliximab with MP and JAK1 inhibitors with steroids have also shown promising response rates, but adverse events profiles have been less favorable.

The management of refractory cGVHD typically involves one of three approved medications: ruxolitinib, ibrutinib, or belumosudil. Ruxolitinib has shown an overall response rate of 40%, with a tolerable adverse events profile. Ibrutinib has shown substantial efficacy in children, with an overall response rate of 77% and a favorable adverse event profile. Belumosudil has shown comparable response rates with an overall response rate of 77%, but with more frequent severe adverse events including pneumonias, hypertension, and hyperglycemia. Other novel options have been explored, with particular attention to tyrosine kinase inhibitors, such as ibrutinib and nilotinib, which have shown promising results in clinical trials. Tyrosine kinase inhibitors have also been studied in combination with other immunotherapies, with some promising results.

Axatilimab, a CSF-1R inhibitor suppressing tissue fibrosis has also shown efficacy against SR-cGVHD with ORR of 82%, 12-months FFS of 77% and favorable toxicity profile in a phase I/II clinical trial. More studies would be beneficial to confirm efficacy and safety of the drug.

Other immunosuppressive drugs, such as rituximab and low-dose IL-2, have also shown efficacy and tolerable adverse event profiles. The hedgehog signaling pathway inhibitor sonidegib and the immunomodulating drug pomalidomide showed less promising results, but further investigation is needed. In summary, larger studies are needed to confirm the efficacy of these novel treatments for cGVHD.

There is a need for more treatment options for SR-cGVHD. FDA-approved options include ruxolitinib and belumosudil for adults and ibrutinib for children. Ibrutinib has shown comparable response rates in adults and children, suggesting underutilization in adults. Nilotinib has also shown comparable response rates to ruxolitinib with a favorable safety profile. Other immunomodulating therapies, including rituximab, low-dose IL-2, and pomalidomide, have shown efficacy similar to approved modalities. Rituximab had a high response rate with a favorable safety profile, but larger studies are needed. Low-dose IL-2 has shown significant response rates and favorable toxicity in children, for whom there is an unmet need. Pomalidomide has shown efficacy similar to ruxolitinib with tolerable adverse events, suggesting it may be effective for further investigation. All the data on the novel drugs in the treatment of chronic SR-GVHD is summarized in Table 2.

## 4. Conclusion

Various novel medications under current investigation could enrich the landscape of acute and chronic SR-GVHD treatment. Monoclonal antibodies, along with other immunosuppressive therapies including pentostatin and itacitinib, showed responses comparable to those of approved therapies, warranting further investigation. Tyrosine kinase inhibitors alone and in combination with other therapies, as well as other immunomodulators such as rituximab and pomalidomide, showed efficacy in adult patient populations, whereas low-dose IL-2 showed promising results in children. Overall, these studies highlight the need for larger studies and increased utilization of novel therapies to improve patient outcomes and resolve existing demand for novel treatment options.

## Figures and Tables

**Table 1:** Novel drugs in the treatment of SR-aGVHD.

Author	Study type	Drug	Mechanism of action	Treatment regimen	ORR	CR	Survival	Grade 3-4 AEs
Jagasia et al.	Prospective, multicenter, open-label, single-cohort, phase II	Ruxolitinib	JAK1 and JAK2 kinase inhibitor	5 mg BID with an increase to 10 mg BID	73.2%	56.3%	MS of 7.6 months	Thrombocytopenia (61%); anemia (45%); infections (40%); neutropenia (40%); hemorrhage (20%); fatigue (14%); elevated transaminases (14%); edema (13%); hypertension (13%); thrombosis (11%); dyspnea (7%); diarrhea (7%); headache (4%); and rash (3%)

Bordigoni et al.	Prospective, single-center, open-label, multicohort, phase II	Daclizumab	IL-2R*α* antagonists	1 mg/kg on days 1, 4, 8, 15, and 22	90.1%	68.8%	4-year EFS 54.6%	38.7% CMV reactivation

Groth et al.	Prospective, single-arm, phase I/II for safety and efficacy	CD3/CD7-IT	Antibodies to CD3/CD7 antigens	Four doses 4 mg/m^2^	60% on day 28	50%	6-months OS 60%	Grade 3: thrombocytopenia (15%), neutropenia (5%), elevated bilirubin (10%), myopathy (5%), microangiopathy (5%), hypoalbuminemia (5%)
							2-year OS 35%	Grade 4: thrombocytopenia (25%)

Socié et al.	Phase III, randomized, open-label, multicenter	Inolimomab	Monoclonal antibody to CD25	Days 1–8 = 0.3 mg/kg/d IV for induction phase and 0.2 mg/kg/d IV for maintenance	NR	NR	1-year OS 28.5%	At least one grade 3, 4, or 5 = 100% in treatment group; 98% in control group
				If CR on day 9, maintenance injection on days 9–28				

Mehta et al.	Prospective study	Vedolizumab	Monoclonal antibody which inhibits interaction between a4b7 on T-cells with MAdCAM-1 on endothelium	IV infusion at a dose of 300 mg at 0, 2, and 6 weeks, and then every 8 weeks depending on the response	25%	20%	35% at 6 months	Infections, liver enzymes elevation

Bacigalupo et al.	Analysis of 2 prospective studies	Begelomab	Monoclonal antibody to CD26	From 2 to 4.5 mg/m^2^/day	75% and 61%	11% and 12%	50% and 30% at 1 year	Group I: bronchopneumonia (25%), dyspnea (16.6%)
								Group II: bacterial sepsis, convulsions, acute respiratory failure, multiorgan failure, *E. coli* infection with renal failure (0.06%, 1 patient)

Bolaños-Meade et al.	Single-center, phase I dose-escalation study	Pentostatin	Nucleoside inhibitor	1 to 4 mg/m^2^/d for 3 days	76%	63%	MS 85 days	Thrombocytopenia 4%, late infections 9%

Benito et al.	Pilot trial for toxicity and efficacy	Sirolimus	mTOR inhibitor	19% of patients 15 mg/m^2^ oral loading dose day 1 + 5 mg/m^2^/d for 13 days	57%	24%	400-day OS 28%	Myelosuppression 9%, seizures 9%
				33% given 5 mg/m^2^/day for 14 days without loading dose				
				48% received 4 mg/m^2^/day for 14 days without loading dose				

Carpenter et al.	Multicenter, single-arm, phase II	Visilizumab	Monoclonal antibody to CD25 and CD3	First dose 3 mg/m^2^; second dose if recurrence in 2–6 weeks	32%	14%	180-day OS 32%	Infusion reaction (4.5%)
							310-day OS 20.5%	
							MS 539 days	

Ho et al.	Single-center, multi-cohort, prospective, phase I	Denileukin diftitox	Cytotoxicity against IL-2 receptors	23% of patients 9 *μ*g/kg on days 1 and 15	71%	50%	6.3–26.4 months OS 33%	Hepatic transaminitis (4%); infusion reaction (6%); acute renal failure (4%); cardiac tamponade (4%); pulmonary embolism (4%); sepsis (11%)
				60% of patients 9 *μ*g/kg IV on days 1, 3, 5, 15, 17, and 19 (maximum tolerated dose)			MS 7.2 months	
				17% of patients given 9 *μ*g/kg IV days 1–5 and 15–19				

Schroeder et al.	Open-label, parallel-cohort, multicenter, phase I	Itacitinib	JAK1-inhibitor	200 mg once daily + corticosteroids or 300 mg once daily + corticosteroids	75% in 200 mg group	37.5% in 200 mg group	6-month OS 58.6%	200 mg group vs. 300 mg group: diarrhea (28.6% vs. 13.3%), edema (0% vs. 1%); abdominal pain (14.3% vs. 6.7%), hypokalemia (28.6% vs. 20.0%), hyperglycemia (21.4% vs. 26.7%), fatigue (28.6% vs. 0%), decreased appetite (14.3% vs. 6.7%), tachycardia (14.3% vs. 0%), hypoalbuminemia (21.4% vs. 6.7%), hypophosphatemia (21.4% vs. 13.3%), nausea (0% vs. 6.7%), and vomiting (0% vs. 6.7%)
					66.7% in 300 mg	22.2% in 300 mg	12-month OS 48.3%	

Marcondes et al.	Phase I/II open-label single-center study,	Alpha-1 antitripsin	Serine protease inhibitor with anti-inflammatory properties	Cohort 1: 90 mg/kg on day 1; 30 mg/kg/day on days 3, 5, 7, 9, 11, 13, and 15. Cohort 2: 90 mg/kg on day 1; 60 mg/kg/day	66.6%	33.3%	50% at >104 to >820 days	None

ORR = overall response rate; CR = complete response; AEs = adverse events; BID = twice daily; MS = median survival; OS = overall survival; EFS = event-free survival; NR = not reported; CG = control group; IL-2R*α* = IL-2 receptor *α*.

**Table 2:** A summary of the novel drugs in the treatment of SR-cGVHD.

Author	Study type	Drug	Mechanism of action	Treatment regimen	ORR	CR	Survival	Grade 3-4 AEs
Zeiser et al.	Multicenter, randomize, open-label phase 3 trial	Ruxolitinib	JAK1 and JAK2 kinase inhibitor	10 mg BID	40%	NR	NR	Thrombocytopenia (33%)
								Anemia (30%)
								CMV infection (26%)

Carpenter et al.	Open-label, multicenter, international phase 1/2 study	Ibrutinib	Bruton tyrosine kinase inhibitor	<12 years: Once daily from 120 mg/m^2^ to 240 mg/m^2;^ patients older than 12 years received 420 mg	77% in R/R group	4% in R/R group	18-months OS 91% in R/R group	64% overall; pyrexia (8%),
								Hypoxia (7%),
								Neutropenia (7%),
								Osteonecrosis (7%),
								Stomatitis (7%),
								ALT elevation (5%),
								Hypokalemia (5%),
								Pneumothorax (5%)

Cutler et al.	Phase 2 randomized multicenter registration trial	Belumosudil	ROCK2 inhibitor	200 mg daily or 200 mg BID	ORR 74% in 200 mg daily group; 77% in 200 mg twice daily group	6% in 200 mg daily group; 3% in 200 mg twice daily group	89% in 2 years	54% overall; pneumonia (8%), hypertension (6%), hyperglycemia (5%), anemia (3%), neutropenia (2%)
Noriko et al.	Multicenter, open label, single-arm study	Ibrutinib	Bruton tyrosine kinase inhibitor	420 mg once daily or 280 mg if concomitant voriconazole	73.7%	10.5%	Not reached	78.9% overall;
								Pneumonia 21.1%
								Cellulitis 15.8%
								Stomatitis 10.5%
								Decreased platelet count 10.5%

Chen et al.	Phase 1/2 clinical trial	Nilotinib	Tyrosine kinase inhibitor	From 200 mg to 400 mg twice daily up to the maximum tolerated dose	46%	0%	NR	Aspiration pneumonia (3%)
								Hyponatremia (3%)
								Hypophosphatemia (10%)
								Influenza B (3%)
								Joint stiffness (3%)
								Lipase elevation (7%)
								LFTs elevation (3%)
								Muscle cramping (3%)
								Myocardial infarction (3%)
								Neutropenia (3%)
								Shingles (3%)
								Pancreatitis (3%)
								*P. jiroveci* pneumonia (3%)
								URI (3%)

Kitko et al.	Phase I/II open-label study	Axatilimab	Monoclonal antibody to CSF-1R	3 mg/kg every four week	82%	NR	12 months FFS was 77%	20% overall;
								Hypertension (4%),
								CPK increase (4%),
								Pneumonia (3%),
								AKI (2%),
								AST increase (2%),
								GGT increase (2%),
								Lipase increase (2%),
								Fever (2%)

DeFilipp et al.	Phase 1 clinical trial	Sonidegib	Hedgehog signaling pathway inhibitor	Up to 12 cycles of 28 days: 200 mg/day (dose level 1), 400 mg/day (dose level 2), and 600 mg/day (dose level 3)	47%	0%	NR	100% overall;
								Arthralgia (18%),
								Abdominal pain (18%)
								Myalgia (18%)
								Back pain (12%)
								Headache (12%)
								Hypercalcemia (12%)
								Single events (6%): anemia, cardiac arrest, chest pain, congestive heart failure, diarrhea, hypertension, hypotension, port infection, skin discoloration, small bowel obstruction, and superior vena cava syndrome

Cutler et al.	Open label phase 1/2 trial	Rituximab	Monoclonal antibody to CD20	38 cycles: 375 mg/m^2^ per week for 4 weeks	70%	10%	NR	43% overall
								Infectious diarrhea (14%)
								Viral conjunctivitis (5%)
								Hepatitis B reactivation (5%)
								Septic arthritis (5%)
								GI hemorrhage (5%)
								Acute infusion reaction (5%)

Whangbo et al.	Single center phase 1 clinical trial	IL-2	Enhancement of regulatory T-cell expansion	Children: 0.33 × 106 IU/m^2^; adults: 0.67 × 106 IU/m^2^ daily	82% in pediatric patients	0%	In 12 months	Pediatric AE: hypophosphatemia (9%)
					Adults: 28.5%		Pediatric patients: 91%	Hypokalemia (9%)
							Adults: 90%	Adult AE:
								Transaminitis (20%)
								Infection (10%)

Curtis et al.	Randomized phase 2 trial	Pomalidomide	Immune modulator	0.5 mg per day orally or 2 mg per day; maximum dose was 2 mg per day	47%	0%	NR	Lymphocytopenia (28%)
								Pneumonia (19%)
								Hypophosphatemia (15%)
								Fatigue (19%)
								Maculopapular rash (3%)
								Skin infection (3%)
								Anemia (6%)
								Limb edema (3%)
								Hypotension (9%)
								Increased ALT (6%)
								Diarrhea (6%)
								Hypertension (3%)
								Leukocytopenia (3%)

ORR = overall response rate; CR = complete response; AEs = adverse events; BID = twice daily; R/R = relapsed/refractory; NR = not reported; ROCK2 = Rho-associated coiled-coil–containing protein kinase 2; CPK = creatinine phosphokinase; AST = aspartate aminotransferase; GGT = gamma-glutamyl transferase; AKI = acute kidney injury.

## Additional Points

*Highlights*. (i) Various novel treatments are under investigation for the treatment of acute and chronic steroid-refractory graft-versus-host disease. (ii) Ruxolitinib remains the only FDA-approved treatment option for steroid-refractory acute graft-versus-host disease whereas ruxolitinib, ibrutinib, and belumosudilare approved for the treatment of chronic steroid-refractory acute graft-versus-host disease. (iii) Monoclonal antibodies and immunosuppressive therapies including pentostatin, itacitinib, rituximab, and pomalidomide, showed promising efficacy warranting further investigation.
